# High Protein Diet Contributes to Insulin Resistance in Mice via Shaping Gut Microbiota

**DOI:** 10.3390/microorganisms13061329

**Published:** 2025-06-07

**Authors:** Yuhui Li, Tiantian Shao, Yating Cao, Jigang Zhang, Anqi Wang, Yichen Shi, Yehao Liu

**Affiliations:** 1 School of Biology, Food and Environment, Hefei University, Hefei 230601, China; liyuhui@hfuu.edu.cn (Y.L.); 18036375235@163.com (T.S.); cyt7chloe@163.com (Y.C.); zhangjg@hfuu.edu.cn (J.Z.); waq1507448262@163.com (A.W.); 2 Department of Hygiene Inspection and Quarantine, School of Public Health, Anhui Medical University, Hefei 230032, China; 2113090215@stu.ahmu.edu.cn

**Keywords:** high protein diet, insulin resistance, gut microbiota, trimethylamine

## Abstract

Insulin resistance (IR) is a risk factor for various diseases. Diet plays a crucial role in the development of IR. The high-protein diet (HPD) is gaining popularity for its weight control benefit. However, some types of protein can be metabolized by gut microbiota into trimethylamine (TMA), subsequently oxidized into trimethylamine N-oxide (TMAO) in the liver. However, the underlying mechanism of HPD-induced IR remains unclear. In this study, we firstly investigated whether the HPD can induce IR. Next, we examined liver function and the signaling pathways involved in IR. At last, we detected changes in the composition and function of gut microbiota, particularly concerning TMA production. Our results demonstrated that the HPD induces IR and liver injury, 41% higher TMA concentration than in the control group. Transcriptome results confirmed that insulin-related pathways were enriched in the HPD group, especially the *Insrr* gene, which regulates insulin action through its receptor, was downregulated. Disrupted gut microbiota, dominated by 65.0% of Firmicutes, which have high potential in TMA production. Moreover, several amino acid metabolism pathways closely linked to IR were enriched in the HPD group. These findings highlight the need for careful dietary management, as the HPD can induce IR and liver injury, with gut microbiota playing a key role in TMA production.

## 1. Introduction

The high-protein diet (HPD) has gained popularity among obese individuals for its potential in weight control and fat mass reduction. Mettler et al. (2010) have reported that the HPD can preserve lean body mass during weight loss [1]. Several underlying mechanisms for the weight control effects of the HPD have been explored, including enhanced satiety, reduced secretion of orexigenic hormones, and improved glucose homeostasis [2,3,4]. However, some negative effects of the HPD have been documented. For instance, when combined with a Western diet, the HPD may exacerbate metabolic diseases by increasing the acid load on the kidneys [5], elevating the risk of calcium stone formation [6], and promoting hepatic gluconeogenesis [7]. An excess protein intake may be converted into glucose through gluconeogenesis or ketone bodies [8,9], potentially disturbing glucose homeostasis. However, whether the HPD increases the risk of insulin resistance (IR) remains unclear.

Many high protein foods, such as eggs, red meat, and fish, contain choline, carnitine, and betaine, which can be metabolize into trimethylamine (TMA) by gut microbiota [10]. TMA is then oxidized into trimethylamine N-oxide (TMAO) in the liver. Elevated TMAO levels have been associated with chronic diseases, including chronic kidney disease, cardiovascular disease (CVD), and diabetes [11,12,13]. Although the HPD has been linked to diabetes [9,13,14], the precise mechanism by which the insulin signaling pathway is disrupted by TMAO and the role of gut microbiota in this process are not well understood. For this purpose, it is necessary to detect the role of gut microbiota in TMA production and its linkage to IR.

Gut microbiota plays a vital role in food metabolism, nutrient provision, and the production of metabolites such as short-chain fatty acids and TMA, which influence host health [15,16]. The diet is one of the most significant factors shaping gut microbiota composition and function. For instance, a high-fat diet enriches Firmicutes, resulting in a high Firmicutes/Bacteroidetes ratio [17], while the HPD enriches Firmicutes and Proteobacteria [18]. Given that TMA production is mediated by gut microbiota, identifying key taxa responsible for TMA production is critical for optimizing dietary patterns. Moreover, efforts should be made to restore the gut microbiota through supplementing probiotics or/and prebiotics, and to define the upper limitation of protein intake in the diet that does not cause IR.

This study aimed to (1) determine the association between the HPD and IR, (2) identify the insulin signaling pathways impacted by TMAO, and (3) investigate changes in gut microbiota composition and function to identify key taxa involved in TMA production.

## 2. Materials and Methods

### 2.1. Animals

Female mice at 7–8 weeks of age were obtained from Beijing Vital River Laboratories (Beijing, China) and housed at Anhui Medical University’s animal center. The mice were acclimated and divided into two groups: the control group (n = 10), which was fed standard chow (AIN93G, 20% energy from protein), and the HPD group (n = 10), which received a high-protein diet (Modified AIN93G with 50% energy from protein). All AIN93G and modified AIN93G were purchased from Jiangsu Xietong Pharmaceutical Bio-Engineering Co., Ltd. (Nanjing, China). The detailed diet composition is listed in Table S1. All mice were housed under pathogen free conditions with a 12 h light and 12 h dark cycle for 24 weeks.

At end of the experiment, mice were killed by intraperitoneal injection of sodium pentobarbital. Blood samples were collected by cardiac puncture, moved to sodium citrate coated tubes, and centrifuged at 8000 rpm at 4 °C for 5 min, and plasma was stored at −80 °C before use. Liver samples were dissected, weighed, and snap frozen in liquid nitrogen and stored at −80 °C. Fresh fecal samples were collected and sent to the Personalbio Technology Co., Ltd. (Shanghai, China) for extracting genomic DNA and 16S rRNA gene analysis.

### 2.2. Ethics Approval

All animal experiments involving in animal handling and management were in accordance with the Laboratory Animal Guide for Care (National Institutes of Health, Publication No. 8523, reviewed in 2011) and the ARRIVE (Animal Research: Reporting of In Vivo Experiments) guidelines, and were approved by the Animal Ethics Committee of Anhui Medical University (LLSC20230605).

### 2.3. The Measurements of TMA in Fecal Samples and TMAO in Plasma Samples

Fecal supernatant samples were used to measure TMA concentration. Briefly, a total of 100 µL of the fecal supernatant was mixed with 300 µL of 80 mM 2,4′-dibromoacetophenone (DBA) in acetonitrile. The mixture was heated at 70 °C for 60 min, then cooled down to 4 °C. Next, the mixture was evaporated using nitrogen at room temperature. After that, 300 µL of ultrapure water was added and vortexed for 5 min. After centrifuging at 15,000× *g* for 5 min, the supernatant was obtained and used for the determination of TMA using an Agilent 7100 series CE instrument (Agilent Technologies, Santa Clara, CA, USA) equipped with a UV-VIS diode array detector.

Plasma TMAO levels were measured using a commercial ELISA kit purchased from Shanghai Tongwei Biotechnology Co., Ltd. (Shanghai, China).

### 2.4. Evaluation of Liver Function

Liver samples were excised and processed according to the manufacturer’s protocol to evaluate liver function. Alanine transaminase (ALT), aspartate transaminase (AST), and alkaline phosphatase (ALP) concentrations were measured using commercial kits from Nanjing Jiancheng Biotechnology Co., Ltd. (Nanjing, China).

### 2.5. Determination of Glucose Tolerance

Glucose tolerance test was conducted at the endpoint of the dietary intervention. After fasting for 12 h, blood samples were collected to measure fasting blood glucose (FBG) using a blood glucose meter. Each mouse was administered with 0.1 mL of a 10 g/mL glucose solution intragastrically, and blood glucose levels were measured at specific time points to construct the area under the oral glucose tolerance test curve (AUC_OGTT_).

### 2.6. Insulin Resistance Test

After a 12 h fast, FBG was measured, and the mice were injected intraperitoneally with 0.75 IU/kg of insulin. Blood glucose was measured at various time points, and the insulin tolerance curve was drawn by calculating the area under the curve.

### 2.7. Transcriptome Analysis of the Liver

Liver samples were collected, and total RNA was extracted using a Total RNA Extractor Kit (Sangon Company, Shanghai, China) following the manufacturer’s instructions. Qualified samples were sent to Personalbio Technology Co., Ltd. for transcriptome sequencing and bioinformatics analysis.

### 2.8. The Validation of DEGs Using Quantitative Real-Time PCR

To validate the DEGs, quantitative real-time PCR (qPCR) was conducted with SYBR Green in a Roche LightCycler 96 thermal cycler (Roche, Basel, Switzerland) under the following conditions: 3 min at 95 °C, followed by 40 cycles of 30 s at 92 °C, 20 s at 60 °C and 30 s at 72 °C. Those selected DEGs included *IL-6*, *PCK1*, *GLUT-2*, *G6PC*, and *Insrr*. The house keeping gene GAPDH was selected for normalization of cDNA amounts. Four replicates were conducted for each sample, and the 2^ΔΔCt^ method was used to calculate the relative level of gene expression. The primer sequences of genes are listed in Table S2.

### 2.9. Metagenomic Analysis of Fecal Samples

Fecal samples were collected, and genomic DNA was extracted using the QIAamp Fast DNA Stool Mini Kit (QIAGEN Company, Hilden, Germany) according to the manufacturer’s instructions. All qualified samples were sent to Personalbio Technology Co., Ltd. for metagenomic DNA library construction and subsequent bioinformatic analysis.

### 2.10. Statistical Analysis

Data were analyzed using GraphPad Prism 9.5. All values are expressed as mean ± standard error. Statistical significance was set at *p* < 0.05.

## 3. Results

### 3.1. The Effects of an HPD on TMA Level in the Gut and TMAO Level in Plasma

**Figure 1:**
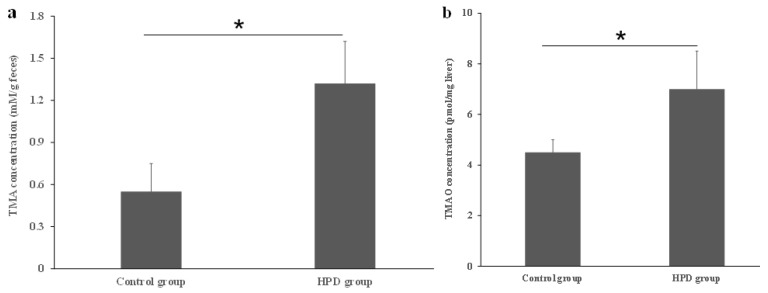
Effects of an HPD on the level of TMA and TMAO, respectively. The TMA level in the gut (**a**), TMAO level in plasma (**b**). * *p* < 0.05.

Dietary proteins contain choline, carnitine, and betaine, which serve as substrates for the production of TMA by gut microbiota. To confirm whether the HPD leads to an increased TMA level in the gut, we measured TMA concentrations in fecal samples. As shown in Figure 1a, the TMA level in the HPD group was 41% higher than in the control group. Next, we evaluated whether the elevated TMA level in the gut resulted in an increased TMAO level in the plasma. Indeed, the plasma TMAO level in the HPD group was 32% higher than in the control group (Figure 1b). These findings suggest that an HPD significantly elevates the plasma TMAO level, which poses a potential health risk.

### 3.2. The Impact of an HPD on Liver Function and Body Weight

**Figure 2:**
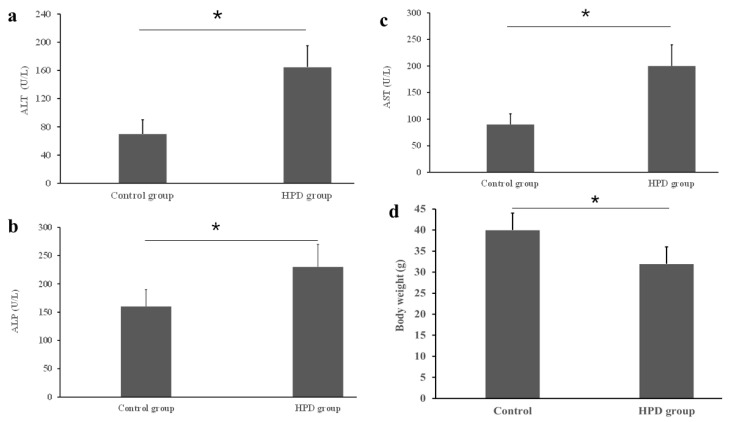
Effects of an HPD on liver function evaluated by the activities of serum ATL (**a**), ALP (**b**), and AST (**c**) in the HPD group and control group. Body weight at the endpoint (**d**). * *p* < 0.05.

An HPD led to the production of excess TMA, which was subsequently oxidized into TMAO in the liver. Given that a high level of TMAO is associated with chronic diseases, particularly with liver dysfunction [19], it was necessary to evaluate the diet’s impact on liver function. Using commercial kits, we measured key liver enzymes and found that the ALT level was significantly higher in the HPD group compared to the control group (Figure 2a). Similar patterns were observed for AST and ALP (Figure 2b,c). Interestingly, statistically significant difference in body weight was observed between the two diet groups, the body weight of the HPD group was lighter by 18.6% than that of control group (Figure 2d). These results suggest that an HPD induces liver dysfunction and the decrease of body weight.

### 3.3. The Effects of an HPD on Blood Glucose and Insulin Levels

**Figure 3:**
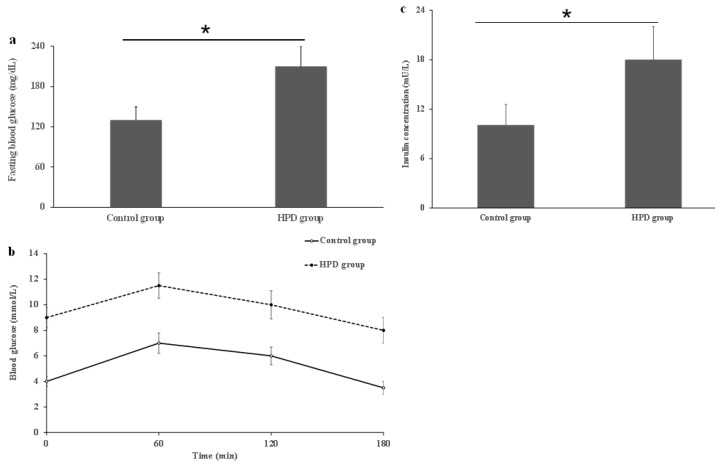
The comparison of fasting blood glucose (**a**), OGTT (**b**), and serum insulin (**c**) in the HPD group and control group. * *p* < 0.05.

To investigate whether the HPD can induce IR in mice, we first compared IR indicators in the two groups. After measuring the levels of serum FBG, OGTT, and insulin, we observed that the HPD group exhibited a significant increase in FBG level compared to the control group (Figure 3a). Next, we also observed that the HPD group showed a gradual increase in the blood glucose level after glucose feeding and reached its maximum value at 1 h, staying at high level even after 2 h post-glucose feeding (Figure 3b), indicating that glucose intolerance was induced. Finally, the HPD group displayed severe IR, evidenced by the significant elevation in the fasting serum insulin level (Figure 3c). These results suggest that an HPD induces IR in mice.

### 3.4. Hepatic Transcriptome Analysis

The liver, where TMA is converted to TMAO, is known to suffer from the harmful effects of excessive TMAO. Therefore, we conducted a transcriptome analysis to assess the impact of an HPD on liver function at the gene expression level. After RNA sequencing, we obtained an average of 540.06 ± 35.46 million clean reads, with over 93% mapped to the mouse reference genome, indicating the reliability of our RNA-sequencing data. We identified approximately 14,730 and 14,620 differentially expressed genes (DEGs) in the HPD and control groups, respectively. Of these, 261 and 151 genes were uniquely expressed in each group, while 14,469 genes were commonly expressed. Interestingly, the *Insrr* gene, which regulates the action of insulin upon binding to its specific receptor, was downregulated in the HPD group.

**Figure 4:**
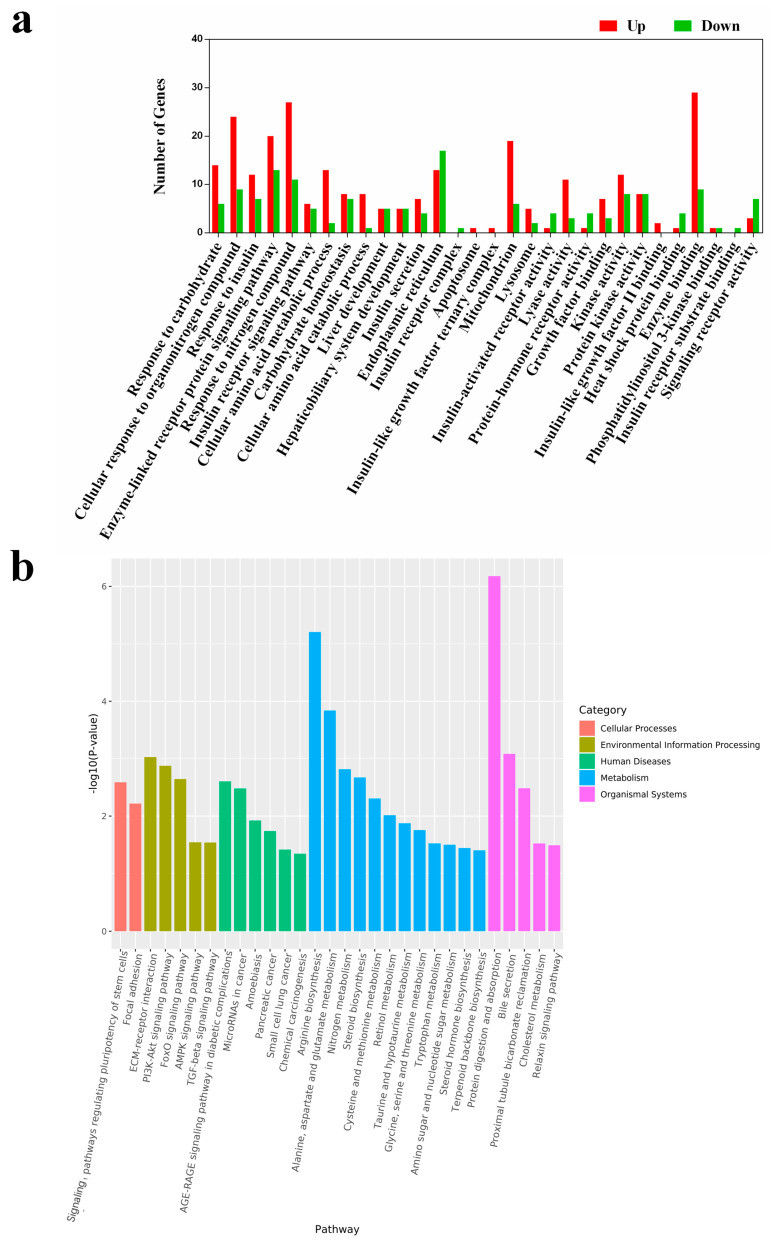
GO enrichment of HP diet induced DEGs (**a**). These DEGs were enriched in 30 sections of the 3 main GO categories. Differentially expressed genes (DEGs) analysis was conducted using the DESeq R package (version 3.6.1). Genes with *p* < 0.05 and |log2 (fold change)| > 1 were considered as DEGs. All these DEGs were subjected to the analysis of GO and KEGG enrichment. Up indicates upregulated terms, and down indicates downregulated terms. A histogram of the KEGG pathway enrichment annotations of the DEGs between control and HPD-fed mice (**b**). *X*-axis indicates functional pathways and *Y*-axis indicates statistical significance.

To understand the biological implications of these DEGs, we performed gene annotation and functional analysis using the Gene Ontology (GO) and KEGG databases. According to the results of GO analysis (Figure 4a), 30 upregulated categories were identified across the three main GO categories (Cellular Component, Biological Process, and Molecular Function). There were 16 DEGs involved in insulin response in the HPD group, including seven in insulin receptor signaling pathways, six in insulin secretion, one in insulin receptor activity, and two in insulin-like growth factor II binding. Interestingly, several DEGs related to the insulin receptor complex and insulin receptor substrate binding were uniquely expressed in the control group.

KEGG enrichment analysis revealed that the DEGs were involved in 30 pathways (Figure 4b). Notably, several pathways, including the AGE-RAGE signaling pathway in diabetic complications, FoxO signaling pathway, nitrogen metabolism, PI3K-Akt signaling pathway, and protein digestion and absorption, were enriched and have been previously linked to insulin-related signaling and protein metabolism [20,21,22]. To verify the reliability of the transcriptome analysis, the expression level of several IR-related DEGs were verified by qPCR. As shown in Figure S1, the expression of the selected genes in qPCR was consistent with that in the transcriptome analysis. These findings indicate that an HPD can impact multiple physiological pathways, particularly those involving insulin signaling.

### 3.5. Taxonomic Analysis of Gut Microbiota Between the Groups

**Figure 5:**
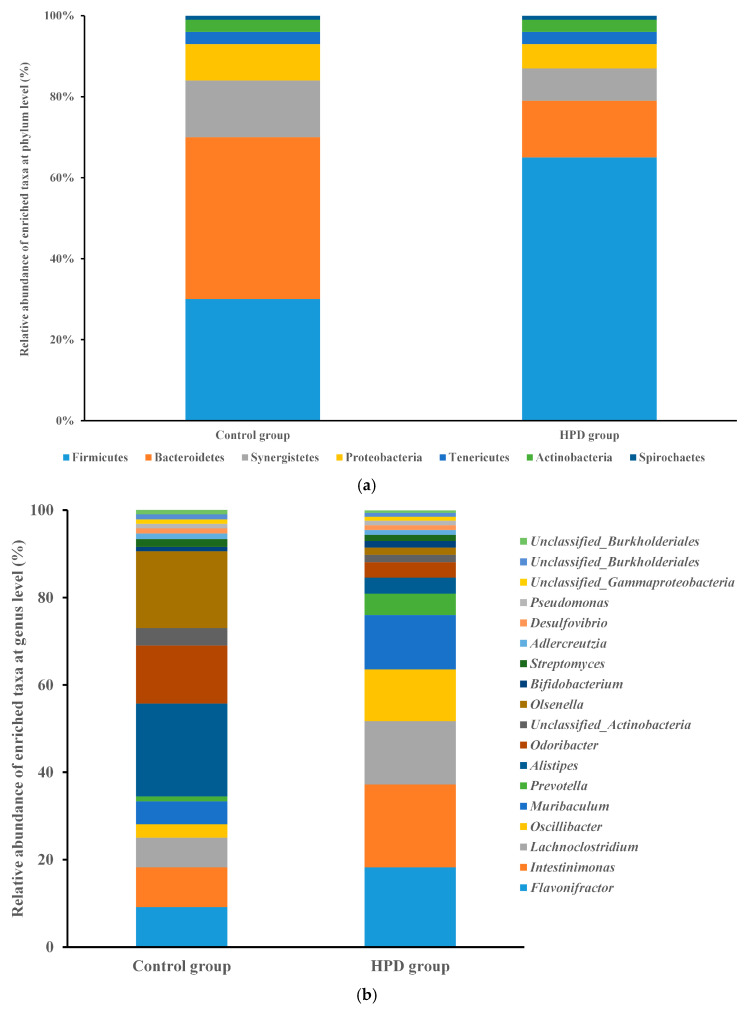
The comparison of the relative abundance of predominant taxa at the phylum level ((**a**), up) and genus level ((**b**), down) in the HPD group and control group.

Given that gut microbiota metabolizes dietary protein into TMA, we sought to investigate the changes in gut microbiota composition and function induced by HPD. After sequencing 10 samples, we obtained 2.4 billion high-quality reads, which were combined to form 533,109 contigs and 941 metagenome-assembled genomes (MAGs). Taxonomic analysis identified 150 bacterial phyla and 2723 genera. Firmicutes were the most abundant phylum in the HPD group, with a relative abundance of 65.0%, followed by the genera *Flavonifractor* (18.2%), *Intestinimonas* (19.0%), *Lachnoclostridium* (14.5%), and *Oscillibacter* (11.9%). In contrast, Bacteroidetes were the most abundant phylum in the control group (40%), with *Alistipes* (21.3%), *Odoribacter* (13.3%), and *Flavonifractor* (9.1%) being the most common genera (Figure 5a,b).

### 3.6. Differences in Gut Microbiota Composition Between the Groups

To compare the gut microbiota composition between the two groups, we first assessed α-diversity (within-sample diversity). The HPD group exhibited lower diversity than the control group, as reflected by the Shannon index (Figure S2 from Supplementary File). Additionally, β-diversity analysis revealed a distinct separation between the two groups along the principal component 1 (PC1), which accounted for 27.9% of the variation, and the principal component 2 (PC2), which accounted for 26.3% (Figure S3 from Supplementary File).

**Figure 6:**
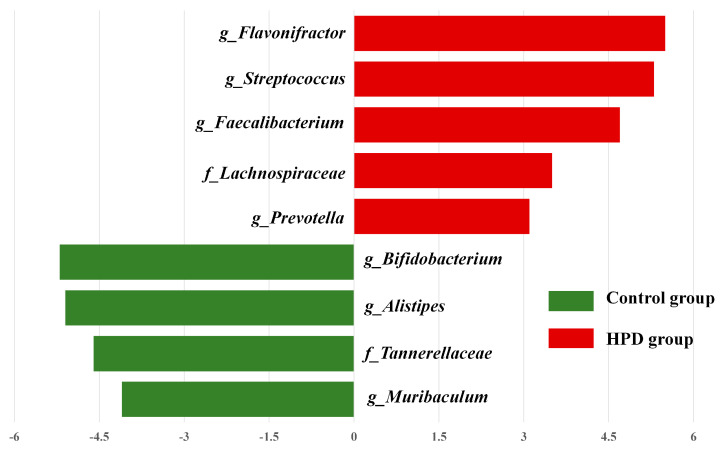
The most enriched taxa in the HPD group and control group, which was determined through LDA score according to linear discriminant analysis effect size (LEfSe) analysis. Green bars indicate that taxa are enriched in the control, and red bars indicate that taxa are enriched in the HPD group.

LEfSe analysis identified significant differences in the abundance of four bacterial genera (*p* < 0.05, LDA > 2) between the HPD and control groups. The enriched genera in the HPD group included *Flavonifractor*, *Streptococcus*, *Faecalibacterium*, and *Lachnoclostridium* (Figure 6), which belong to the phylum Firmicutes.

### 3.7. Functional Changes in Gut Microbiota Between the Groups

**Figure 7:**
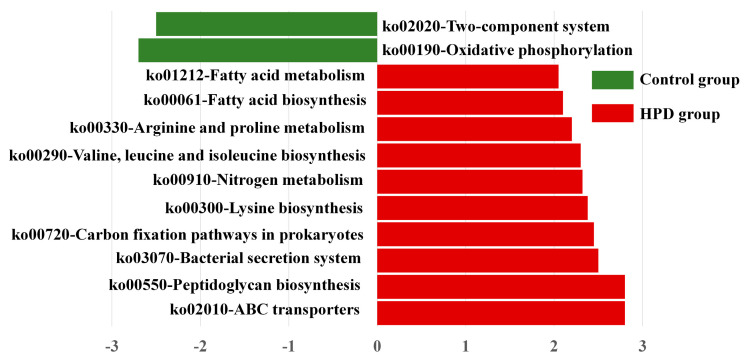
LEfSe plot of enriched KEGG pathways in the HPD group and control group.

To explore whether the HPD affected the function of gut microbiota, we conducted LEfSe analysis of KEGG pathways. As shown in Figure 7, we identified 10 pathways enriched in the HPD group, including ko02010 (ABC transporters), which are involved in protein and carbohydrate metabolism. In contrast, two KEGG pathways were enriched in the control group: ko02020 (two-component system) and ko00190 (oxidative phosphorylation). These findings suggest that an HPD alters the functional capacity of gut microbiota, enhancing its ability to metabolize proteins and carbohydrates.

### 3.8. Genes Involved in Protein and Carbohydrate Metabolism

**Figure 8:**
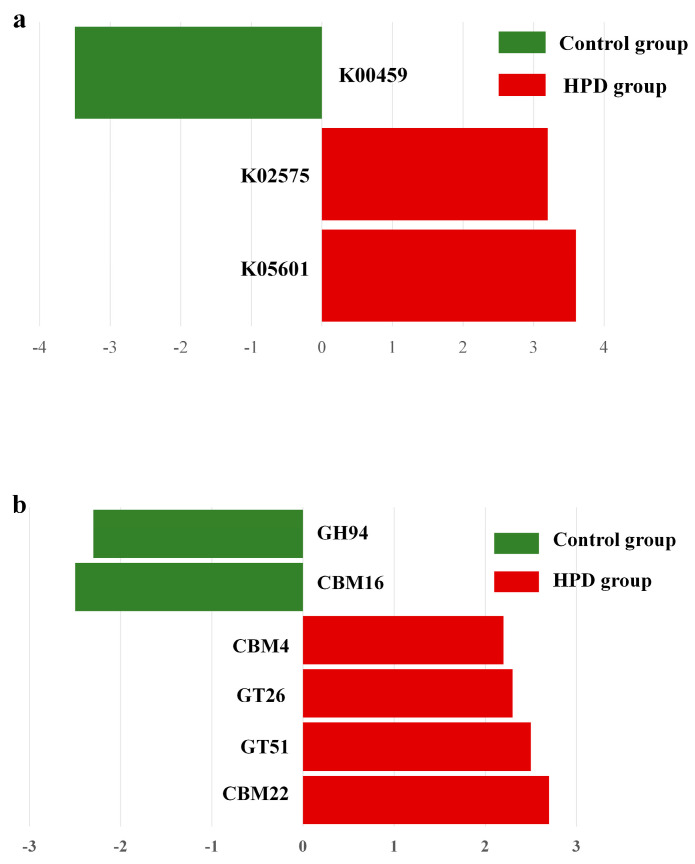
LEfSe plot of enriched KEGG genes involving in nitrogen metabolism (**a**) and carbohydrate metabolism (**b**) in the HPD group and control group. The function of KEGG genes in nitrogen metabolism: K00459 for nitronate monooxygenase, K02575 for nitrate/nitrite transporter, and K05601 for hydroxylamine reductase. The function of KEGG genes in carbohydrate metabolism: GH94 for glycosyltransferase, CBM16 for carbohydrate Binding Module Family 16, CBM4 for carbohydrate-Binding Module Family 4, GT26 for β-1,4-galactosyltransferase, GT51 for Peptidoglycan glycosyltransferase, and CBM22 for carbohydrate Binding Module Family 22.

Given our interest in the genes involved in protein and carbohydrate metabolism, we analyzed the relevant enzymes encoded by the gut microbiota. In the nitrogen metabolism pathway (Figure 8a), two KEGG genes (K02575 and K05601) were enriched in the HPD group, while one KEGG gene (K00459) was enriched in the control group. Further analysis identified four bacterial species (*Eubacterium pyruvativorans*, *Coprococcus catus*, *Megasphaera elsdenii*, and *Prevotella copri*) that exhibited significantly higher gene abundances in the HPD group. Additionally, carbohydrate-active enzymes (CAZymes) analysis revealed an enrichment of CBM4, GT26, GT51, and CBM22 families in the HPD group, while only GH94 and CBM16 families were enriched in the control group (Figure 8b).

## 4. Discussion

In this study, we demonstrated that an HPD can induce IR in the liver through elevated level of TMAO derived from gut microbiota-produced TMA. The dysfunction of gut microbiota plays a crucial role in this process, likely by impairing the binding of insulin to its receptor. These findings suggest that careful consideration is needed when evaluating the benefits of the HPD, particularly in terms of body weight control.

We compared the transcriptomic differences in the liver between the two groups and found that most DEGs and pathways were involved in carbohydrate, insulin, nitrogen compound responses, and insulin-related pathways. In evaluating the impact of excessive TMAO on the liver, we focused on gene-level changes. GO enrichment analysis revealed that DEGs responsible for modulating insulin receptor complex and insulin receptor substrate binding were completely inhibited in the HPD group. The insulin receptor complex, after binding to insulin, is internalized and processed by target cells [23]. It has been reported that individuals with IR have a dysfunctional insulin receptor complex, and the absence of gene expression may lead to an incomplete structure of the insulin receptor complex, increasing the risk of IR in liver cells [24]. Insulin receptor substrate plays a pivotal role in the insulin signaling cascade, and impairment of this pathway can contribute to IR [25]. Our findings align with this understanding. While several IR-related KEGG pathways were enriched, it remains challenging to pinpoint the exact pathway most critical at the gene level. Based on GO enrichment analysis, we infer that the insulin resistance-related KEGG pathways involved in insulin binding to its receptor or receptor substrate will likely be impaired in the HPD group.

To further understand the differences in gut microbiota composition and function between the two groups, we performed metagenomic sequencing and taxonomic analysis. The results indicated significant differences in gut microbiota composition, with the HPD group exhibiting significantly lower diversity compared to the control group. Phyla such as Firmicutes, Proteobacteria, and Actinobacteria were enriched in the HPD group, while Bacteroidetes was inhibited. Several studies have indicated that these enriched phyla, particularly Firmicutes, are potential TMA producers. The high Firmicutes/Bacteroidetes ratio in the HPD group has been positively associated with plasma TMAO concentration [26]. Additionally, LEfSe analysis revealed that several opportunistic pathogens and TMA-producing taxa, such as Prevotella, Chloroplast, and Firmicutes, were enriched in the HPD group, while probiotics like *Bifidobacteriaceae*, *Prevotellaceae*, and *Rhodospirillales* were enriched only in the control group. It has been reported that individuals with high plasma TMAO levels tend to be dominated by Prevotella, while those with lower TMAO levels are dominated by Bacteroides [27,28]. Various studies have also indicated that TMA-producing gut microbiota are more prevalent within the Firmicutes phylum and less so in the Bacteroidetes phylum [26,29]. Moreover, a cross-sectional analysis reported a positive association between plasma TMAO levels and the abundance of Proteobacteria in older participants [30]. Conversely, probiotics such as *Bifidobacteriaceae* and *Lactobacillus* were diminished in the HPD group. Since the HPD has a low carbon-to-nitrogen (C/N) ratio and its degradation products can raise intestinal pH levels, this may suppress the growth of beneficial probiotics.

Furthermore, several genera enriched in the HPD group were involved in protein and carbohydrate metabolism. Previous research has shown that certain amino acids, such as isoleucine, valine, tyrosine, and alanine, are positively associated with homeostasis model assessment (HOMA)-IR levels in overweight and obese participants [31]. Our metagenomic data also indicated that the KEGG pathways responsible for the biosynthesis of these amino acids were enriched in the HPD group, increasing the risk of IR. Additionally, a study reported that gut microbial carbohydrate metabolism increases the risk of IR in mouse models [32]. We also observed an upregulation of carbohydrate-active enzymes (CAZymes) in the HPD group, suggesting a higher carbohydrate degrading activity by the gut microbiota.

## 5. Conclusions

In this study, we first evaluated the impact of an HPD on liver function, particularly insulin sensitivity. We then explored the potential mechanisms behind the development of IR in the liver due to the HPD. Lastly, we compared the differences in gut microbiota composition and function between mice fed a high-protein diet and those on a normal diet. While a greater number of enriched taxa were observed in the control group, four species in the HPD group harbored genes involved in the degradation of proteins and carbohydrates, suggesting a high potential for TMA production. Our findings indicate that further evaluation is required to fully understand the benefits and potential risks of the HPD, particularly with regard to body weight control and metabolic health.

There are several limitations in this study. Firstly, no causal relationship between gut microbiota, TMA/TMAO, and insulin resistance has been confirmed, although the association between them was observed. Secondly, although an insulin receptor binding gene, *Insrr*, was identified using transcriptome analysis, receptor-binding or structural assays were not carried out. Thirdly, no insulin-related pathway was confirmed. These limitations alert us to pay more attention to uncover the mechanism of insulin resistance caused by high protein diet.

## Data Availability

The original data presented in the study is included in this article; any inquiries can be directed to the corresponding author.

## Supplementary Materials

The following supporting information can be downloaded at: https://www.mdpi.com/article/10.3390/microorganisms13061329/s1, Figure S1: The validation of DEGs using qPCR. Figure S2: The comparison of Shannon index between Control group and HPD group; Figure S3: The difference in the composition of gut microbiota between control group and HPD group based on the data of 16S rRNA; Table S1: Diet composition; Table S2: The primer sequence pairs used in qPCR.

## Abbreviations

IR, insulin resistance; HPD, high-protein diet; TMA, trimethylamine; TMAO, trimethylamine N-oxide; AIN 93G, American Institute of Nutrition 93G; ELISA, enzyme-linked immunosorbent assays; ALT, alanine transaminase; AST, aspartate transaminase; ALP, alkaline phosphatase; FBG, fasting blood glucose; AUC_OGTT_, the area under the oral glucose tolerance test curve; OGTT, oral glucose tolerance test; DEGs, differentially expressed genes; GO, gene ontology; KEGG, Kyoto Encyclopedia of Genes and Genomes; AGE-RAGE, advanced glycation end-products receptor for AGE
