# Supplementary material for: High Protein Diet Contributes to Insulin Resistance in Mice via Shaping Gut Microbiota
Source: Microorganisms. 2025 Jun 7;13(6):1329. doi: 10.3390/microorganisms13061329 (PMC12195126; doi:10.3390/microorganisms13061329)
Supplement: Supplementary file 1 [file microorganisms-13-01329-s001.zip › microorganisms-3643917-supplementary.pdf]

Table S1. Diet composition

	Control diet	High protein diet
Protein (kcal%)	20	50
Casein	200 g kg^{-1}	540 g kg^{-1}
Fat (kcal%)	12	12
Carbohydrate (kcal%)	68	38
Fiber (%)	4.7	4.7
Choline (mg/kg)	1.67	1.67
Vitamin Mix V10001 (g/kg)	1	1
Mineral Mix S10026B (g/kg)	50	50

Table S2. The primer sequence pairs used in qPCR

Gene	Sequence of the primers (5’-3’)
IL-6	F: GGCAATTCTGATTGTATG
	R: CTCTGGCTTTGTCTTTCT
PCK1	F: CATAACGGTCTGGACTTCTCTGC
	R: GAATGGGATGACATACATGGTGCG
GLUT-2	F: TGGCTCGGGGACAAACTT
	R: AGCAATGATGAGGGCGTGT
G6PC	F: ATGAACATTCTCCATGACTTTGGG
	R: GACAGGGAACTGCTTTATTATAGG
Insrr	F: TATATCGCTCTGGACTTCTCTGC
	R: GACTCCGATGACATACATGGTGCG
GAPDH	F: GTGGCAAAGTGGAGATTGTTG
	R: AGTCTTCTGGGTGGCAGTGAT

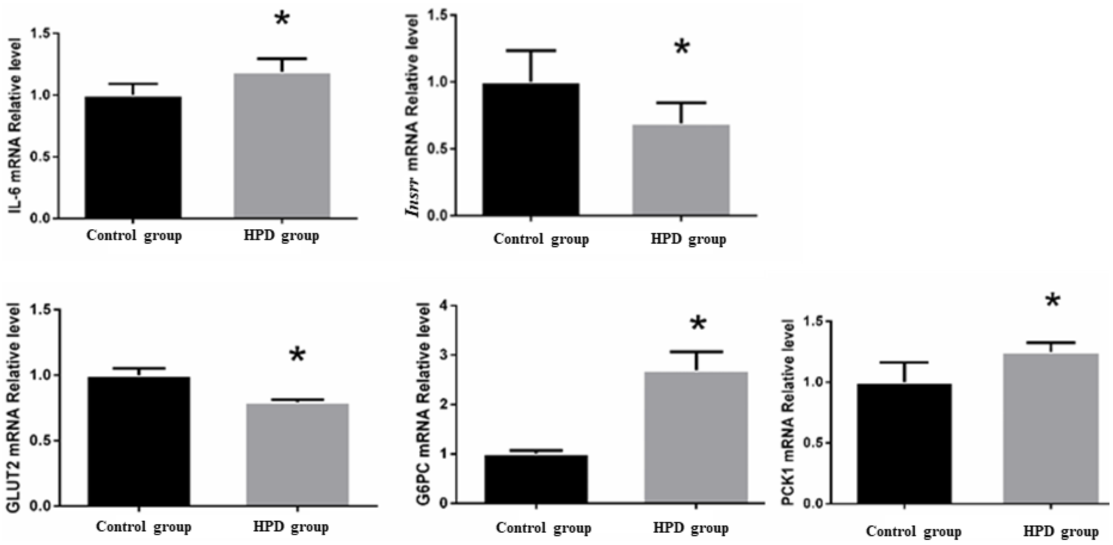

Figure S1. The validation of DEGs using qPCR. Results are expressed as mean ± SEM. *$p < 0.05$

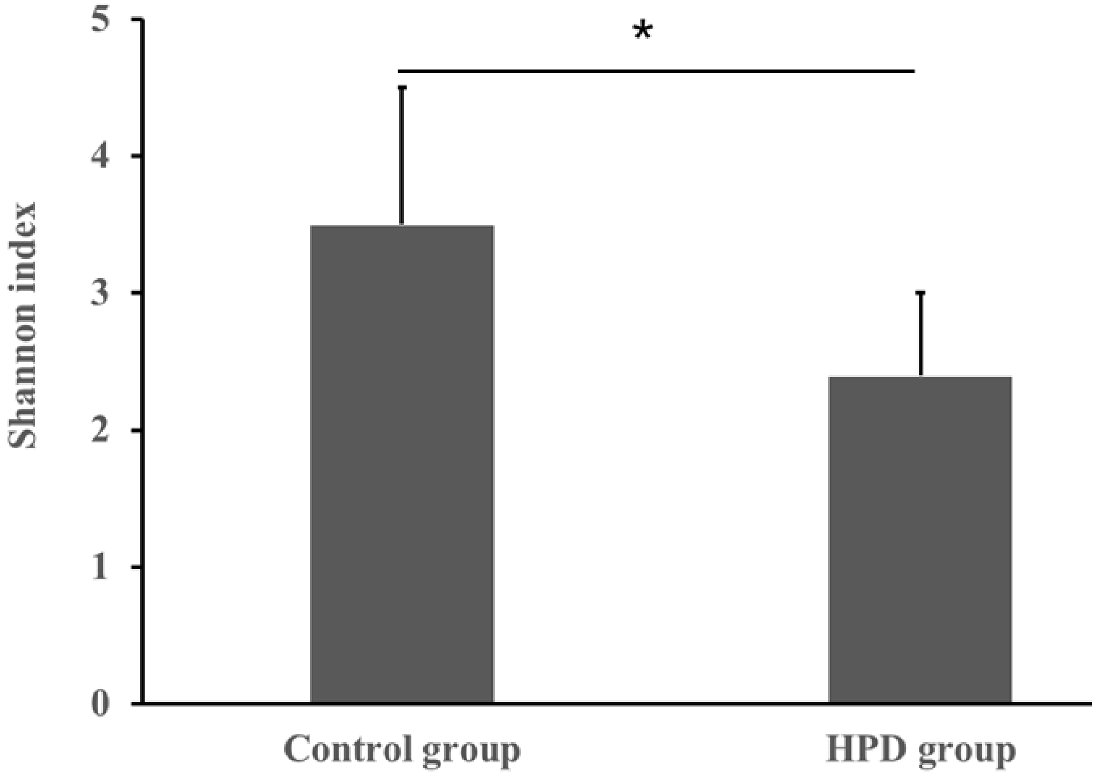

Figure S2. The comparison of Shannon index between Control group and HPD group.
*$p < 0.05$

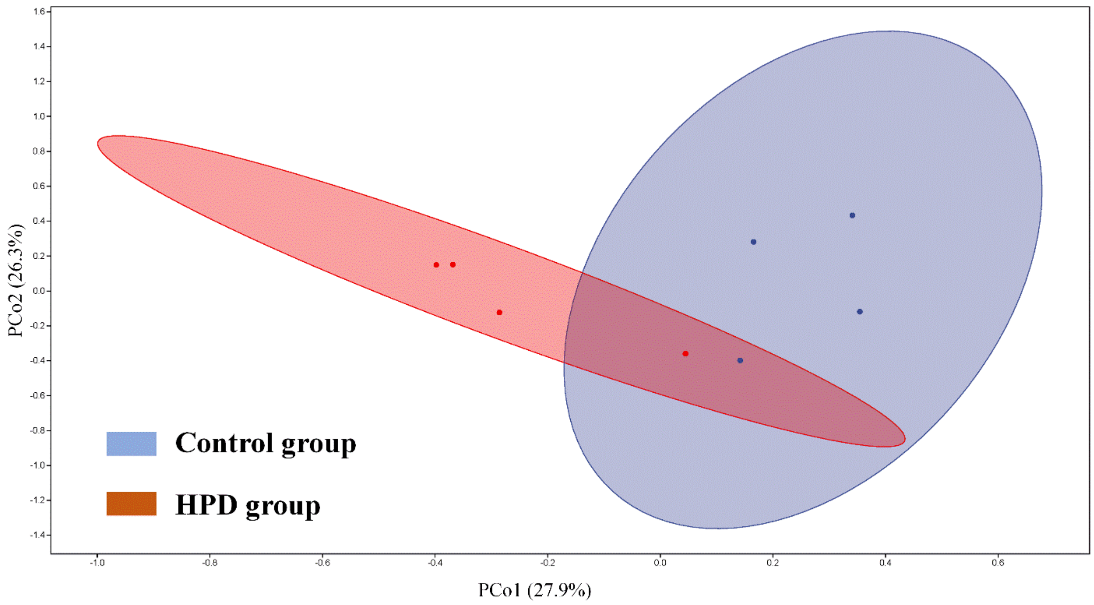

Figure S3. The difference in the composition of gut microbiota between control group and HPD group based on the data of 16S rRNA. Principal coordinate analysis (PCoA) with unweighted UniFrac distance shows that the gut microbiota of HPD separate from that of HPD group in the first axis.
